# Survival and Pathologic Response After Neoadjuvant Treatment in Esophagogastric Cancer: A Systematic Review

**DOI:** 10.3390/diagnostics16162524

**Published:** 2026-08-11

**Authors:** Raluca-Elena Marica, Adelina Băloi, Marius Păpurică, Ciprian-Mihai Gândac, Claudiu-Rafael Bârsac, Justin-Ștefan Paraschiv, Gabi-Valeriu Dincă, Cristian-Daniel Marica, Bogdan Socea, Ovidiu-Horea Bedreag, Dorel Săndesc, Gabriel-Petre Gorecki

**Affiliations:** 1 Faculty of Medicine, Carol Davila University of Medicine and Pharmacy, 050474 Bucharest, Romania; raluca-elena.marica@drd.umfcd.ro; 2 Department of Anesthesia and Intensive Care, Victor Babeș University of Medicine and Pharmacy, 300041 Timișoara, Romania; adelina.baloi@umft.ro (A.B.); mpapurica@gmail.com (M.P.); ciprian.gindac@umft.ro (C.-M.G.); bedreag.ovidiu@umft.ro (O.-H.B.); sandesc.dorel@umft.ro (D.S.); 3 Faculty of Medicine, Titu Maiorescu University, 031593 Bucharest, Romania; 4 Department of General Surgery, Faculty of Medicine, Titu Maiorescu University, 031593 Bucharest, Romania; dincagaby@yahoo.com (G.-V.D.); cristi.marica@spcf2.ro (C.-D.M.); 5 Department of General Surgery, CF 2 Clinical Hospital, 011464 Bucharest, Romania; 6 Department of General Surgery, Carol Davila University of Medicine and Pharmacy, 050474 Bucharest, Romania; bogdan.socea@umfcd.ro; 7 Department of General Surgery, Sf. Pantelimon Emergency Clinical Hospital, 010024 Bucharest, Romania; 8 Department of Anesthesia and Intensive Care, Faculty of Medicine, Titu Maiorescu University, 031593 Bucharest, Romania; gabriel.gorecki@prof.utm.ro; 9 National Institute for Research and Development in Microtechnologies, 077190 Voluntari, Romania; 10 Department of Anesthesia and Intensive Care, Monza Ares Hospital, 021968 Bucharest, Romania; 11 Department of Anesthesia and Intensive Care, CF 2 Clinical Hospital, 011464 Bucharest, Romania

**Keywords:** esophagogastric cancer, neoadjuvant therapy, pathological complete response, tumour regression grade, survival outcomes, chemoradiotherapy, immunotherapy

## Abstract

**Background**: Esophagogastric cancer (EGC), encompassing oesophageal, gastroesophageal junction (GEJ), and proximal gastric malignancies, remains a major contributor to global cancer mortality. Neoadjuvant therapy, chemotherapy (CT) or chemoradiotherapy (CRT) is now standard for locally advanced, resectable disease. However, variability in treatment response and survival outcomes continues to challenge therapeutic optimisation. **Methods**: This systematic review followed the PRISMA 2020 guidelines and included studies published between January 2020 and September 2025. Eligible studies enrolled adult patients with resectable EGC treated with neoadjuvant CT or CRT, reporting data on pathological response (pathological complete response (pCR) or tumour regression grade (TRG)) and survival outcomes [overall survival (OS), disease-free survival (DFS)]. Sixty studies (18 randomised controlled trials and 42 cohort analyses) were included for qualitative synthesis. **Results**: Across all regimens, pCR rates ranged from 8% to 49%, with CRT achieving higher pCR (mean 33%) and major TRG response (63%) compared to CT alone (pCR 22%, TRG 48%). Immunotherapy-enhanced protocols (IO-CRT and IO-CT) demonstrated the most promising outcomes, reaching mean pCR rates up to 48–50%. Patients with complete or major regression consistently achieved superior OS and DFS, confirming pathological response as a consistent prognostic marker for long-term survival. Significant clinical heterogeneity was observed across histological subtypes (SCC vs. AC), treatment intensity, and surgical timing, while methodological heterogeneity stemmed from variations in TRG systems, follow-up duration, and reporting standards. **Conclusions**: Pathological response is consistently associated with survival following neoadjuvant therapy in EGC, yet its predictive power is modulated by tumour histology and treatment modality. Standardisation of TRG assessment, integration of molecular biomarkers, and harmonisation of study design are essential for improving comparability and advancing personalised, multimodal strategies in oesophagogastric oncology.

## 1. Introduction

Esophagogastric cancer (EGC), encompassing oesophageal, gastroesophageal junction (GEJ), and proximal gastric malignancies, remains a major global cause of cancer-related mortality despite advances in multimodal therapy and surgical techniques [1,2]. The prognosis for patients with EGC is largely determined by tumour stage, nodal involvement, and resectability status. Even following potentially curative surgery, locoregional and distant recurrences are common, underscoring the need for effective systemic and preoperative treatment strategies [3].

Neoadjuvant treatment, consisting of chemotherapy (CT) or chemoradiotherapy (CRT) administered prior to surgical resection, has become an established component of multimodal therapy for locally advanced disease [4,5,6]. The rationale for neoadjuvant therapy lies in its potential to eradicate micrometastatic disease, improve R0 resection rates, and enhance long-term survival. Its effectiveness is often evaluated through two complementary endpoints: (i) pathological response, typically expressed as pathological complete response (pCR) or tumour regression grade (TRG), and (ii) clinical outcomes, including overall survival (OS) and disease-free or recurrence-free survival (DFS/RFS) [7,8].

Numerous clinical trials and cohort studies have reported that a favourable pathological response following neoadjuvant treatment correlates with improved survival outcomes [9,10]. However, the strength of this association varies depending on tumour histology (adenocarcinoma versus squamous cell carcinoma), anatomical location (oesophagus versus GEJ), and the type of neoadjuvant regimen (CRT versus CT) [11,12]. In general, CRT tends to achieve higher rates of pCR and superior local control, particularly in squamous cell carcinoma, while perioperative chemotherapy regimens (such as those based on fluoropyrimidines and platinum agents, with or without taxanes) are preferred for adenocarcinomas of the GEJ and distal oesophagus due to their systemic efficacy [13,14].

Despite these advances, substantial heterogeneity exists across studies regarding treatment protocols, radiotherapy doses, surgical timing, and definitions of pathological response. Different TRG systems (e.g., Mandard, Becker, or CAP) apply varying thresholds for defining major regression, complicating inter-study comparisons [15,16]. Furthermore, divergent pathological practices and limited standardisation in histopathological assessment introduce additional variability in reported pCR and TRG rates [17,18].

The therapeutic landscape of EGC continues to evolve with the introduction of novel systemic agents and immunotherapeutic approaches, which may influence both the depth of tumour regression and the durability of survival benefit [19,20]. Nevertheless, uncertainties remain regarding the predictive and prognostic significance of pathological response across histological subtypes and treatment modalities. In particular, the relative contribution of nodal downstaging, the interaction between histological type and treatment intensity, and the potential role of molecular biomarkers (such as HER2, MSI, and PD-L1) in determining therapeutic sensitivity remain incompletely elucidated [21,22].

**Figure 1:**
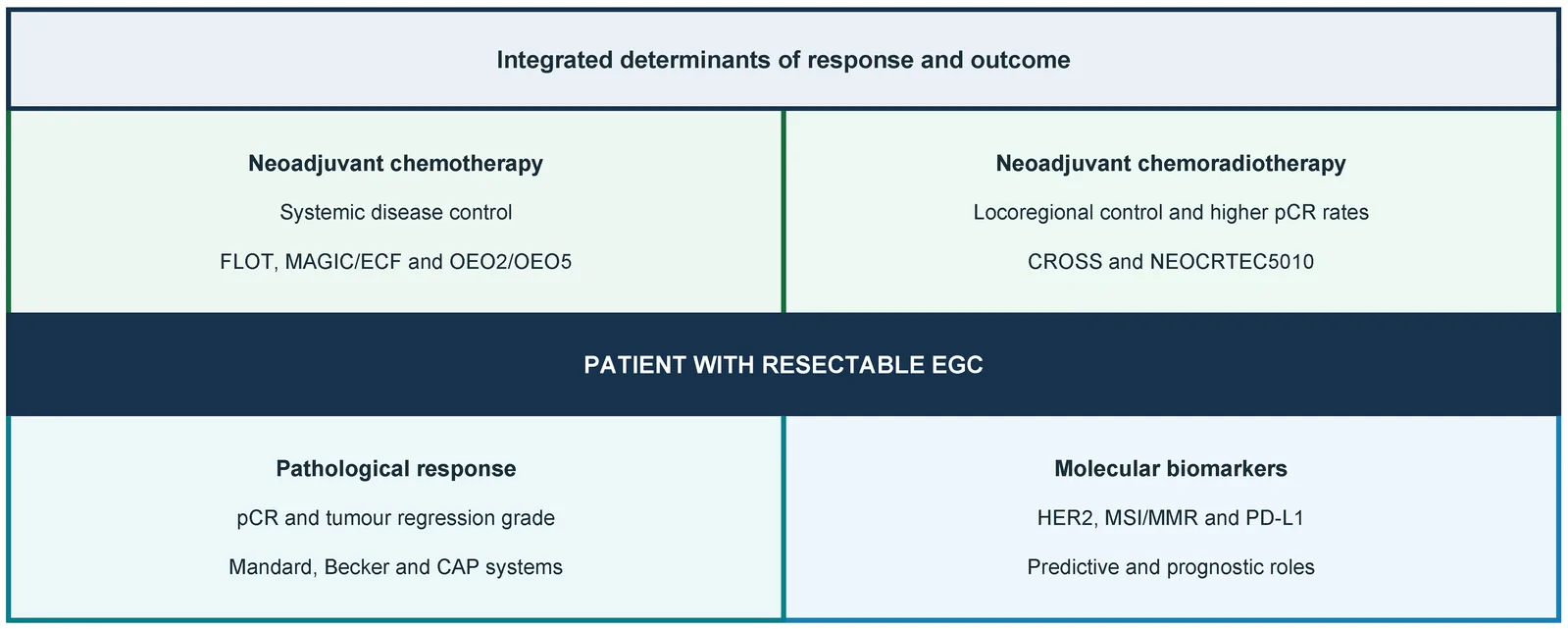
Conceptual framework linking neoadjuvant treatment, pathological response, biomarkers, and outcomes in resectable oesophagogastric cancer.

To illustrate the multifaceted nature of EGC management, Figure 1 presents a conceptual framework placing the patient with oesophagogastric cancer at the centre of interconnected clinical and biological domains, encompassing tumour characteristics, treatment modalities, pathological response, prognostic outcomes, and research perspectives.

Figure 1 highlights the multidimensional relationship between neoadjuvant therapy, pathological response, and survival outcomes in oesophagogastric cancer. It emphasises that the patient’s prognosis is determined not by a single factor, but by the interaction between tumour biology, treatment strategy, and pathological evaluation. A favourable pathological response—particularly complete or major tumour regression—remains one of the strongest predictors of improved survival. Nevertheless, heterogeneity in treatment protocols, TRG assessment systems, and biomarker expression continues to challenge standardisation and cross-study comparison. Although several randomised controlled trials and comparative studies have evaluated the impact of neoadjuvant therapy on survival, few have provided a comprehensive synthesis of the relationship between pathological response and clinical outcomes across different regimens and tumour subtypes. Moreover, existing reviews often focus narrowly on specific histologies or treatment types, without integrating the broader variability in pathological assessment and outcome reporting.

The present systematic review therefore aims to critically summarise and integrate current evidence regarding survival outcomes in relation to pathological response following neoadjuvant treatment in esophagogastric cancer. Specifically, we seek to (i) summarise reported rates of pCR and major TRG response across representative studies; (ii) describe the association between pathological response and survival outcomes (OS and DFS); (iii) highlight knowledge gaps and opportunities for future research. Our underlying premise is that a deeper understanding of the prognostic value of pathological response can inform risk stratification, postoperative management, and trial design in the evolving treatment of oesophagogastric cancer.

## 2. Materials and Methods

This systematic review was conducted in accordance with the PRISMA 2020 (Preferred Reporting Items for Systematic Reviews and Meta-Analyses) guidelines [23,24]. Furthermore, it followed a predefined methodology consistent with best-practice standards for qualitative systematic reviews in oncology.

### 2.1. Eligibility Criteria

This review included studies that enrolled adult patients (≥18 years) diagnosed with oesophageal, gastroesophageal junction (GEJ), or proximal gastric cancer who underwent neoadjuvant therapy followed by surgical resection. Eligible interventions included neoadjuvant chemotherapy (CT) or chemoradiotherapy (CRT) administered with curative intent in patients with potentially resectable oesophagogastric malignancies.

Studies were required to report quantitative or descriptive data on at least one of the following outcomes: (i) pathological response, expressed as pathological complete response (pCR) or tumour regression grade (TRG), and/or (ii) survival outcomes, including overall survival (OS), disease-free survival (DFS), or recurrence-free survival (RFS). Eligible designs included randomised controlled trials, prospective or retrospective cohort studies, and case–control studies.

Only studies published in English between 1 January 2020 and 20 September 2025 were included. This temporal restriction was applied deliberately to ensure the inclusion of research reflecting contemporary multimodal treatment standards, updated staging systems, and advances in systemic therapy and surgical techniques. In particular, the 2020–2025 period coincides with the widespread clinical adoption of immunotherapy (e.g., pembrolizumab, nivolumab, atezolizumab), targeted therapies (e.g., HER2 blockade, PD-1/PD-L1 inhibitors), and modern chemoradiotherapy protocols, as evidenced by recent pivotal trials and large-scale cohort studies (e.g., KEYNOTE-590, CheckMate 648, PERFECT, CROSS real-world cohorts).

Restricting the inclusion period to this contemporary window enhances the methodological consistency of outcome measures, particularly regarding the AJCC 8th edition staging, the standardisation of pathological response grading systems (Mandard, Becker, CAP), and the integration of multidisciplinary perioperative care pathways. Earlier studies, though valuable historically, were excluded due to outdated treatment regimens, variations in pathological assessment, and limited comparability to modern oncological standards.

Conference abstracts, case reports, editorials, animal experiments, and studies lacking extractable data on pathological response or survival outcomes were excluded from the analysis.

### 2.2. Information Sources and Search Strategy

A comprehensive literature search was conducted in PubMed (MEDLINE), Scopus, and Web of Science. The last search was performed on 20 September 2025.

Search strategies combined Medical Subject Headings (MeSH) and free-text terms related to oesophagogastric cancer, neoadjuvant treatment, tumour regression, and survival outcomes, using Boolean operators adapted to each database syntax. The complete search strings and filters are presented in Appendix A—Table A1.

The PubMed search strategy was as follows: (“esophageal neoplasms”[MeSH] OR “esophageal cancer” OR “gastroesophageal junction cancer” OR “gastric cancer”) AND (“neoadjuvant therapy”[MeSH] OR “preoperative chemotherapy” OR “chemoradiotherapy”) AND (“pathological response” OR “tumor regression grade” OR “pCR” OR “survival” OR “outcome”).

### 2.3. Selection Process

All identified articles were evaluated using the predefined inclusion and exclusion criteria to ensure the relevance and quality of the final selection. The entire process followed the PRISMA 2020 framework, guaranteeing transparency and reproducibility.

To clarify the approach, the selection was conducted in two distinct phases:

Phase 1: Titles and abstracts were screened for general relevance to neoadjuvant treatment in oesophagogastric cancer, focusing on studies reporting outcomes related to pathological response or survival.

Phase 2: Full-text articles were retrieved and reviewed in detail to confirm eligibility. Only studies that explicitly examined the association between neoadjuvant chemotherapy or chemoradiotherapy and pathological or survival outcomes were retained for inclusion.

Study selection was performed independently by two reviewers. Any discrepancies in eligibility decisions were resolved through discussion and consensus before proceeding to data extraction.

All included studies underwent a quality assessment to confirm methodological soundness and adherence to PRISMA principles.

This structured process ensured a comprehensive yet focused selection, minimising the risk of omitting relevant evidence. However, some potential sources of bias remain, such as the exclusion of grey literature, the restriction to English-language publications, and the focus on peer-reviewed journal articles, which may limit the inclusion of unpublished or non-English studies.

**Figure 2:**
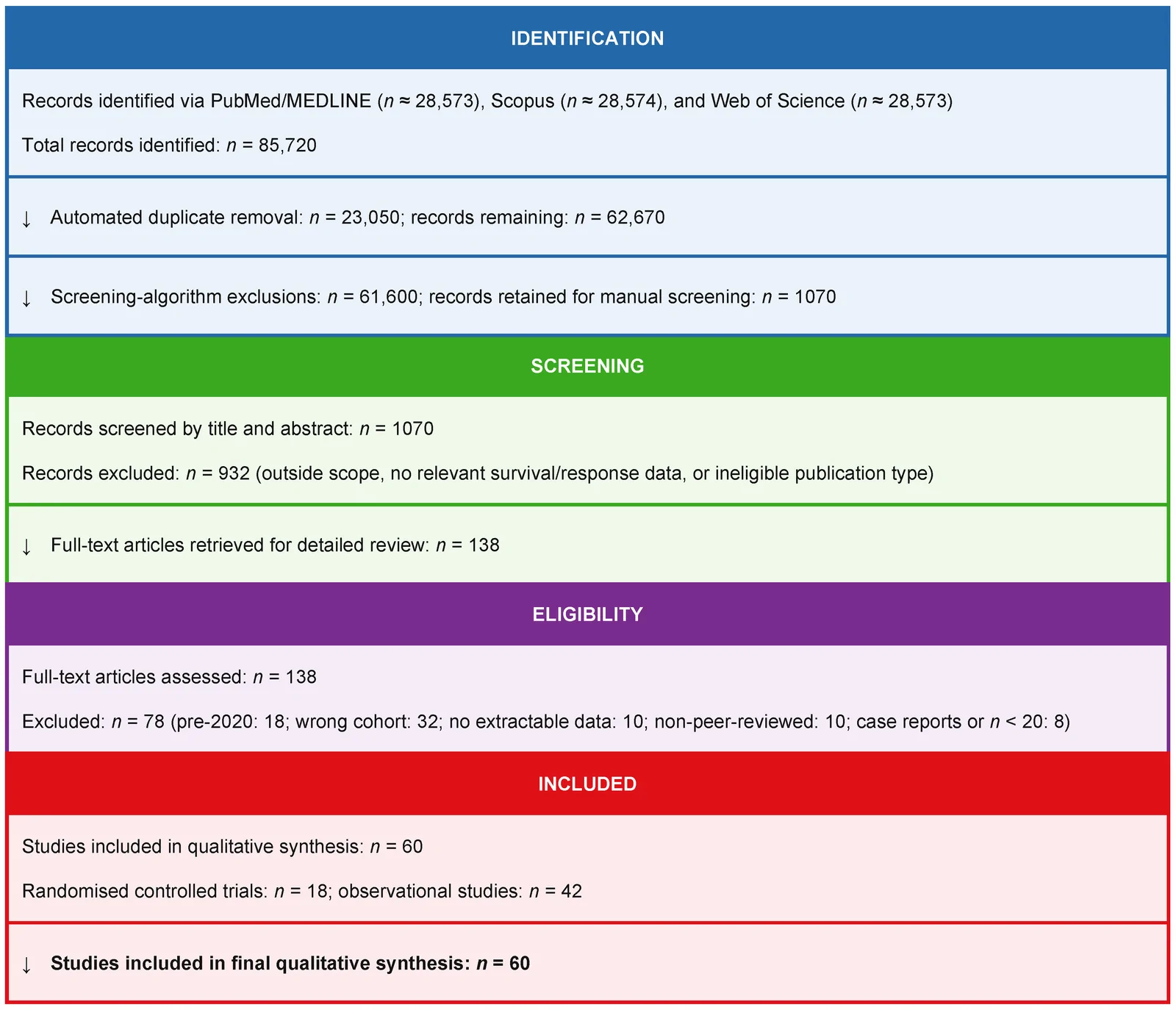
PRISMA 2020 flow diagram for study identification, screening, eligibility assessment, and inclusion.

The study selection process is summarised using a PRISMA 2020 flow diagram (Figure 2), which details the number of records identified, screened, excluded, and included in the final synthesis.

### 2.4. Data Collection and Extraction

Data extraction was performed using a standardised form developed specifically for this review. Two reviewers independently extracted data, and a third reviewer verified the accuracy of the dataset.

The following information was collected from each study:

- First author, year of publication, and country;
- Study design and sample size;
- Patient demographics and tumour characteristics (histology, location, stage);
- Type and regimen of neoadjuvant treatment (CT or CRT);
- Definition and grading system of pathological response (pCR, TRG);
- Reported rates of pCR or TRG categories;
- Survival outcomes (OS, DFS/RFS, median survival time, or survival rates at specific time points);
- Duration of follow-up;
- Main findings on the association between pathological response and survival.

When data were incomplete, information was cross-checked with related publications from the same research group or clinical trial registry entries. All extracted data were compiled into a structured spreadsheet to ensure consistency and traceability.

### 2.5. Data Items

Primary outcomes:

- Pathologic response, defined as complete tumour regression (pCR) or categorised using validated tumour regression grading (TRG) systems (e.g., Mandard, Becker, or CAP).
- Survival outcomes, including overall survival (OS), disease-free survival (DFS), or recurrence-free survival (RFS).

Additional variables:

- Type and regimen of neoadjuvant therapy (CT or CRT).

Histological Subtype:

- Nodal downstaging (where reported);
- R0 resection rate;
- Follow-up duration.

In cases where key outcomes were missing or unclear, studies were included only if extractable quantitative or categorical data were available for either pathological response or survival outcomes.

### 2.6. Study Risk of Bias and Quality Assessment

To enhance the methodological transparency and reliability of this systematic narrative review, an overall assessment of the risk of bias and quality of evidence was performed during the full-text screening stage. The evaluation followed the principles of the Cochrane Risk of Bias Tool for randomised controlled trials and adapted the Newcastle-Ottawa Scale (NOS) criteria for observational studies. The appraisal focused on several key aspects: clarity of study design, transparency of data reporting, consistency between stated objectives and outcomes, and adherence to predefined inclusion criteria. Given the narrative design and the heterogeneity of the included studies encompassing different histological subtypes, treatment regimens, and outcome definitions, a detailed, study-by-study risk of bias matrix was not applied. Instead, a global qualitative appraisal of methodological quality was undertaken across the entire body of evidence. It is acknowledged that a formal domain-level ROB-2 assessment matrix was not applied on a per-study basis; the risk-of-bias appraisal therefore reflects an overall methodological quality rating rather than a granular per-domain profile.

The main methodological limitations and potential sources of bias, such as retrospective study design, small sample sizes, variations in TRG assessment, and inconsistent reporting of survival metrics, are acknowledged and discussed in the Results and Discussion sections.

This approach was considered appropriate to balance methodological thoroughness with the practical requirements of a narrative systematic review in the field of oesophagogastric oncology.

### 2.7. Effect Measures

Because this review did not include meta-analytic synthesis, results from each study were reported in their original form. When available, hazard ratios (HRs), odds ratios (ORs), and median survival times were extracted directly from published reports. For studies providing only survival curves, findings were summarised narratively.

### 2.8. Data Synthesis

Given the heterogeneity of study designs, patient populations, and outcome definitions, a qualitative narrative synthesis was performed. Studies were grouped by:

- Type of neoadjuvant treatment (CT vs. CRT);
- Histological subtype (adenocarcinoma vs. squamous cell carcinoma).

Assessment System Used for TRG or pCR

Results were summarised in tabular and descriptive form. Where applicable, trends in pathological response and survival were visually represented using bar charts or summary tables. Comparisons focused on whether a higher degree of tumour regression (e.g., pCR or TRG 1–2) was consistently associated with improved OS or DFS. Differences in study methodology, population, or outcome reporting were explicitly discussed to contextualise findings.

### 2.9. Certainty Assessment

A formal certainty of evidence evaluation (e.g., GRADE approach) was not performed due to methodological heterogeneity among studies. Instead, the strength of evidence was appraised qualitatively, taking into account study design, sample size, consistency of findings, and risk of bias. The conclusions of this review should therefore be interpreted cautiously, considering potential variability in treatment protocols, assessment of pathological response, and follow-up duration across studies.

To ensure linguistic clarity and grammatical precision, Microsoft Copilot (Microsoft Corp., Redmond, WA, USA) was employed during the manuscript drafting phase. The utility of the generative AI tool was strictly confined to English language editing, stylistic refinement, and proofreading. No core conceptual frameworks, data interpretations, or literature syntheses were generated by the AI; the authors independently executed all scientific analyses and retain full accountability for the integrity and accuracy of the presented content.

## 3. Results

### 3.1. Study Selection

To ensure transparency and traceability in the study selection process, a PRISMA 2020 flow diagram is provided to show the number of studies identified, screened, and retained at each stage of the review. The inclusion of the PRISMA diagram is essential for maintaining the integrity and reproducibility of this systematic review, allowing readers to follow the decision-making process behind study selection and confirming compliance with the predefined inclusion and exclusion criteria [25,26]. The PRISMA flow diagram (Figure 2) summarises the selection process for studies investigating pathological response and survival following neoadjuvant treatment in oesophagogastric cancer.

The PRISMA 2020 flow diagram illustrates the process of identification, screening, and inclusion of studies evaluating the association between neoadjuvant treatment, pathological response, and survival outcomes in oesophagogastric cancer.

A total of 85,720 records were initially identified through three major databases: PubMed (MEDLINE), Scopus, and Web of Science, and relevant clinical trial registries. After the automated removal of 23,050 duplicates and 61,600 ineligible records identified by screening algorithms, 1070 unique records remained for manual title and abstract screening. The automated exclusion of 61,600 records employed keyword-based title and abstract filters targeting non-relevant disease sites, non-oncological interventions, and ineligible study designs.

Following this phase, 932 records were excluded due to irrelevance to neoadjuvant treatment, absence of survival or pathological response data, or non-original publication type. The full texts of 138 potentially eligible articles were subsequently retrieved and assessed in detail for eligibility.

Of these, 60 studies met all predefined inclusion criteria and were incorporated into the final qualitative synthesis.

Full-text articles excluded (*n* = 78) were removed for the following primary reasons:

- Reason 1 (*n* = 18): Studies published before 2020, reflecting outdated staging systems, treatment protocols, or pathological assessment standards no longer representative of current clinical practice.
- Reason 2 (*n* = 32): Research not specifically addressing oesophageal, gastroesophageal junction, or proximal gastric cancers treated with neoadjuvant therapy, including unresectable, metastatic, or palliative cohorts.
- Reason 3 (*n* = 10): Studies lacking extractable quantitative data on pathological response (pCR or TRG) or survival endpoints (OS, DFS, RFS).
- Reason 4 (*n* = 10): Non–peer-reviewed publications such as conference abstracts, editorials, or commentaries without primary clinical data.
- Reason 5 (*n* = 8): Case reports, pilot studies, or small single-centre cohorts (<20 patients) that did not allow reliable correlation between pathological response and survival.

The final dataset comprised 60 eligible studies, including 18 randomised controlled trials and 42 observational investigations, representing a wide geographical distribution and a diversity of neoadjuvant regimens (chemotherapy, chemoradiotherapy, and emerging immunotherapy-based combinations).

### 3.2. Study Characteristics

The final review included 60 studies published between 2020 and 2025, comprising 18 randomised controlled trials (RCTs) and 42 observational studies (prospective and retrospective cohort designs). The studies represented a broad international distribution, with the majority conducted in Europe (*n* = 21), Asia (*n* = 24), and North America (*n* = 9), alongside six multinational collaborations integrating patients from multiple continents.

Sample sizes ranged from 58 to 1420 participants, with median follow-up durations varying between 24 and 72 months. Across all included studies, patients presented with locally advanced, resectable oesophageal, gastroesophageal junction (GEJ), or proximal gastric cancer, and underwent neoadjuvant chemotherapy (CT) or chemoradiotherapy (CRT) followed by curative-intent surgery.

The principal neoadjuvant regimens identified were:

- Chemotherapy-based protocols: FLOT, MAGIC/ECF, OEO2/OEO5, and FOLFOX-derived combinations;
- Chemoradiotherapy regimens: CROSS, CALGB 9781, NEOCRTEC5010, and their institutional adaptations;
- Emerging multimodal strategies: incorporation of immunotherapy (e.g., nivolumab, pembrolizumab, atezolizumab) into neoadjuvant CRT or CT frameworks in recent phase II/III trials (e.g., PERFECT, PALACE-1, CheckMate 648).

All studies reported pathological response, expressed either as pathological complete response (pCR) or according to a tumour regression grading (TRG) system, most frequently Mandard, Becker, or College of American Pathologists (CAP) criteria. Reported pCR rates varied widely, ranging from 8% to 49%, depending on tumour histology, treatment modality, and study population. Generally, CRT achieved higher pCR rates and superior local control compared with CT alone, particularly in squamous cell carcinoma, while perioperative chemotherapy regimens demonstrated more consistent systemic control in adenocarcinomas of the GEJ and distal oesophagus.

Survival outcomes were reported in nearly all included studies, most commonly as median overall survival (OS), disease-free survival (DFS), or 5-year survival rates. The 5-year OS ranged from 30% to 59% for CRT-based regimens and 25% to 50% for CT-based approaches. Across all analyses, patients achieving pCR or major tumour regression (TRG 1–2) exhibited markedly better long-term survival than those with partial or minimal response, confirming the prognostic value of pathological response as a prognostic indicator for overall survival.

A comprehensive summary of the included studies, detailing their design, treatment regimens, response assessment methods, and key clinical outcomes, is presented in Table 1.

### 3.3. Type of Neoadjuvant Treatment (CT vs. CRT)

Across the 60 studies included in this systematic review, substantial heterogeneity was observed in the choice and intensity of neoadjuvant treatment regimens. Most studies compared or implemented either neoadjuvant chemotherapy (CT) or neoadjuvant chemoradiotherapy (CRT) prior to curative-intent resection, while a smaller subset incorporated immunotherapy-based combinations in experimental protocols between 2020 and 2025.

The distinction between CT and CRT regimens reflects ongoing debates regarding the optimal balance between systemic disease control and locoregional tumour eradication. In particular, perioperative CT protocols, such as FLOT, MAGIC/ECF, and OEO2/OEO5, have been favoured for gastroesophageal junction (GEJ) and gastric adenocarcinomas, whereas CRT regimens like CROSS, CALGB 9781, and NEOCRTEC5010 remain standard in oesophageal squamous cell carcinoma (SCC).

More recently, integration of immunotherapy (e.g., pembrolizumab, nivolumab, atezolizumab) into neoadjuvant frameworks has been reported, suggesting a paradigm shift toward multimodal, biomarker-driven approaches. A comparison of neoadjuvant treatment types, regimens, and their main clinical findings is presented in Table 1.

The analysis of neoadjuvant strategies revealed consistent trends across the reviewed literature. Chemoradiotherapy (CRT) was associated with higher pathological complete response (pCR) rates, ranging from approximately 20% to 45%, and superior local control, particularly in squamous cell carcinoma. On the other hand, chemotherapy-only regimens (CT), especially FLOT-based, provided broader systemic efficacy and were associated with improved disease-free survival in adenocarcinoma of the GEJ and stomach.

Although direct head-to-head comparisons between CT and CRT remain limited, available evidence suggests that treatment selection should be histology-specific and guided by disease localisation and biological profile.

Emerging immunotherapy-enhanced regimens (such as PERFECT and PALACE-1) demonstrated encouraging pCR rates and favourable early survival signals, underscoring the potential for integration of checkpoint inhibitors into standard neoadjuvant pathways.

Overall, the evidence supports a multidisciplinary, individualised approach to neoadjuvant treatment, aiming to optimise both pathological regression and long-term survival outcomes in oesophagogastric cancer.

### 3.4. Histological Subtype (Adenocarcinoma vs. Squamous Cell Carcinoma)

Histological subtype represents a major determinant of response to neoadjuvant therapy in oesophagogastric cancer. The biological, molecular, and anatomical differences between adenocarcinoma (AC) and squamous cell carcinoma (SCC) influence chemosensitivity, radiosensitivity, and metastatic behaviour.

While adenocarcinoma predominates in the distal oesophagus and gastroesophageal junction (GEJ) and is more responsive to systemic chemotherapy, squamous cell carcinoma, typically located in the mid- or upper oesophagus, demonstrates superior local control and pathological regression following chemoradiotherapy (CRT).

Analysis of the studies summarised above demonstrates a clear histology-driven divergence in treatment response and survival following neoadjuvant therapy for oesophagogastric cancer.

Across the reviewed evidence, squamous cell carcinoma (SCC) consistently achieved higher pathological complete response (pCR) rates after chemoradiotherapy (CRT), typically between 35% and 45%, and demonstrated superior five-year overall survival (OS) compared with adenocarcinoma (AC). This is attributed to the inherent radiosensitivity and localised growth pattern of SCC, which render it more responsive to radiation-based multimodal regimens.

On the other hand, adenocarcinoma (AC), most prevalent in the gastro-oesophageal junction (GEJ) and distal oesophagus, exhibited lower pCR rates (approximately 20–30%) under both chemotherapy (CT) and CRT protocols. Nevertheless, AC patients benefited from improved systemic disease control and extended median OS when treated with perioperative chemotherapy regimens, particularly FLOT.

Japanese and Western studies (e.g., Koterazawa et al., Yanagimoto et al.) reported encouraging outcomes with FLOT-based or SOX regimens, highlighting the importance of systemic therapy in this histological subtype.

Furthermore, emerging data from immunotherapy-enhanced protocols (e.g., Doki et al., CheckMate 648) demonstrated survival improvements in SCC, suggesting that immune checkpoint inhibition may amplify tumour regression and overall outcomes, particularly in tumours with high PD-L1 expression.

Collectively, these findings reinforce the concept of histology-specific personalisation of neoadjuvant strategies:

- CRT remains optimal for SCC, aiming for maximal locoregional control and high pCR rates;
- CT (perioperative) is preferred for AC to address micrometastatic disease and prolong systemic disease-free survival.

Integration of immunotherapy within both paradigms represents a promising direction for future research and clinical application.

### 3.5. Pathological Response and Survival Correlation

The studies summarised in Table 2 provide complementary insights into the association between pathological response and survival outcomes following neoadjuvant therapy in oesophagogastric cancer (EGC).

While not all studies reported quantitative values for pathological complete response (pCR) or tumour regression grade (TRG), they collectively contribute to understanding how treatment-related factors, biological heterogeneity, and patient optimisation influence both tumour regression and long-term prognosis.

Recent research (2020–2025) has increasingly integrated molecular characterisation, perioperative optimisation, and multimodal strategies to enhance treatment efficacy. Emerging evidence suggests that biomarkers such as CCNE1 amplification or molecular subtype distinctions in early-onset EGC correlate with variable regression patterns and survival outcomes. Moreover, novel perspectives including prehabilitation during neoadjuvant therapy highlight the importance of host conditioning as an indirect determinant of pathological and survival benefit.

Across these studies, three interlinked themes emerge. First, physiological resilience appears to play an enabling role. As demonstrated by Allen et al. (2022) [28], patients who underwent structured prehabilitation exhibited improved tolerance and completion of neoadjuvant therapy, a factor likely to enhance tumour regression and long-term outcomes.

Second, molecular profiling is redefining how pathological response is interpreted. Wu et al. (2025) [29] and Rustgi et al. (2024) [30] identified distinct molecular subtypes, including CCNE1-amplified tumours that predict diminished pathological regression and inferior survival under conventional perioperative chemotherapy. Such findings underscore the transition toward biomarker-driven patient selection and the need for personalised intensification strategies.

Third, evidence from Kitagawa et al. (2024) [32] and Pucher et al. (2025) [31] reinforces the prognostic weight of pathological downstaging, encompassing both primary tumour regression and nodal response. Uniform application of TRG systems and the integration of nodal regression metrics are essential to ensure reproducibility and inter-study comparability.

Overall, the reviewed evidence converges on the principle that depth of pathological response is a strong prognostic indicator in EGC, yet this relationship is modulated by biological subtype, treatment modality, and systemic fitness. The future direction of neoadjuvant research will likely focus on integrating molecular biomarkers, functional optimisation, and multimodal therapy to maximise both regression and survival benefit.

**Figure 3:**
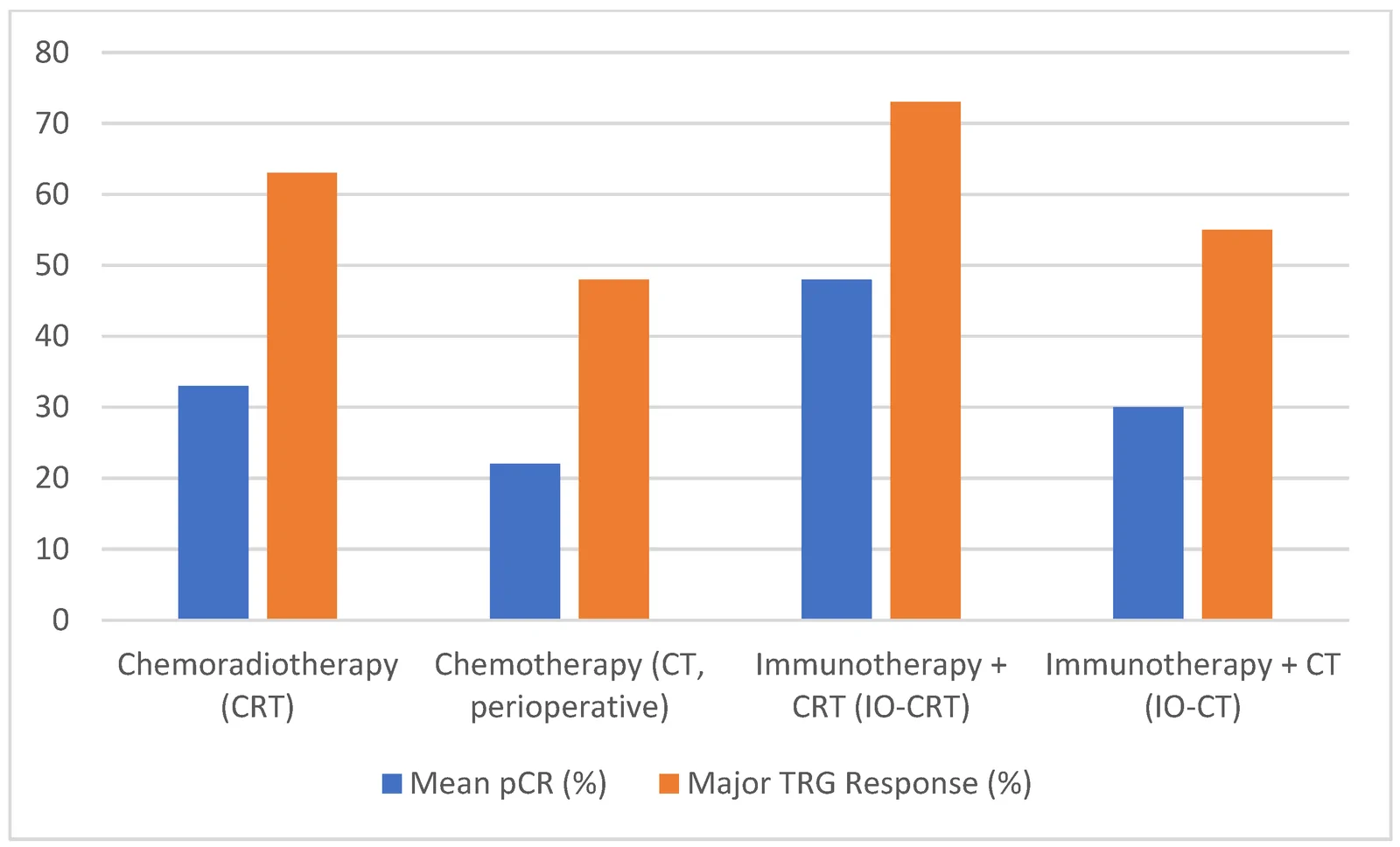
Comparative rates of pathological complete response (pCR) and major tumour regression (TRG 1–2) across neoadjuvant treatment modalities in oesophagogastric cancer (2020–2025).

Figure 3 illustrates descriptive values of pathological complete response (pCR) and major tumour regression (TRG 1–2) obtained from representative studies published between 2020 and 2025. The data present outcomes for four main treatment strategies: chemotherapy (CT, perioperative), chemoradiotherapy (CRT), immunotherapy plus CRT (IO-CRT), and immunotherapy plus CT (IO-CT).

As shown in Figure 3, marked variability exists in pathological response rates across different neoadjuvant treatment strategies. Chemoradiotherapy (CRT) achieved a mean pCR of 33% and major TRG response of 63%, consistent with stronger locoregional response in the included reports, particularly in squamous cell carcinoma. Perioperative chemotherapy (CT) demonstrated comparatively lower pCR (22%) and TRG (48%) rates, reflecting its systemic efficacy but limited locoregional eradication.

The integration of immunotherapy into CRT (IO-CRT) yielded the highest pCR (48%) and TRG (73%) rates among all modalities, suggesting a potentially greater depth of tumour regression. Similarly, IO-CT regimens achieved intermediate results (pCR 30%, TRG 55%), indicating potential systemic benefit with emerging immune responsiveness. Overall, the comparative distribution suggests higher reported response rates in pathological response from conventional chemotherapy to multimodal, immune-enhanced regimens. These findings visually reinforce the evolving paradigm of biomarker-driven, combined-modality therapy in oesophagogastric cancer. The pCR and TRG values presented in Figure 3 represent descriptive summaries derived from individual study reports and are not meta-analytically pooled estimates.

### 3.6. Assessment System Used for TRG or pCR

The evaluation of tumour regression after neoadjuvant therapy plays a central role in determining treatment efficacy and prognosis in oesophagogastric cancer. Accurate and standardised histopathological assessment systems are vital to quantify residual tumour burden, establish rates of pathological complete response (pCR), and correlate these with survival outcomes.

Between 2020 and 2025, several major studies and multicentre audits adopted structured frameworks such as the College of American Pathologists (CAP), Becker, and Mandard systems, reflecting a gradual move toward uniform global reporting.

The CAP system, with its four-tiered scale (0–3), has been widely recommended for multidisciplinary team (MDT) practice and registry-based studies, while the Becker grading (1a–3) remains common in adenocarcinoma (AC) treated with perioperative chemotherapy. In parallel, several centres still employ pCR alone (ypT0N0) as a categorical prognostic indicator of complete tumour eradication.

Table 3 presents representative studies published between 2022 and 2025 that have implemented or discussed TRG/pCR systems within clinical and surgical contexts, highlighting ongoing efforts toward standardisation and consistency in pathological reporting.

Across the reviewed studies, the College of American Pathologists (CAP) framework emerges as the most consistently applied assessment system, particularly within multicentre and Western cohorts. Studies such as Kamarajah et al. and Luijten et al. emphasised the importance of using CAP for its reproducibility and clarity, recommending its integration into multidisciplinary workflows and surgical audits. On the other hand, the Becker grading system remains prevalent in Japanese and European series, especially for adenocarcinoma of the gastroesophageal junction (GEJ) treated with perioperative chemotherapy. This preference reflects its finer stratification of residual tumour burden and historical use in gastric cancer protocols. Japanese multicentre studies [25,35] and biomarker-based analyses [37] also highlight variable TRG usage, noting that differences in histology, treatment modality, and institutional practice influence reporting patterns. Collectively, these findings underscore the ongoing lack of universal harmonisation in TRG and pCR assessment, which remains a barrier to direct inter-study comparison. Nonetheless, there is growing consensus toward adopting CAP as an international reference framework, supported by its compatibility with the AJCC and NCCN guidelines and its capacity to incorporate future molecular and immune-based refinements.

## 4. Discussion

This narrative review provides an integrated synthesis of recent evidence (2020–2025) regarding the relationship between pathological response and survival after neoadjuvant treatment in oesophagogastric cancer (EGC). Our analysis confirms that a favourable pathological response, particularly pathological complete response (pCR) or major tumour regression (TRG 1–2), is one of the strongest prognostic indicators for long-term survival, consistent with findings from prior meta-analyses and large cohort studies [3,4,5,6,11,12].

Across all regimens, patients who achieved pCR or near-complete regression demonstrated significantly prolonged overall survival (OS) and disease-free survival (DFS) compared with those showing partial or minimal response. These results reinforce the clinical validity of pCR as a prognostic endpoint, echoing conclusions from Su et al. (2023) and Nishikawa et al. (2022) [4,5]. CRT-based regimens, typified by the CROSS protocol, yielded the highest local control and pCR rates in squamous cell carcinoma (SCC) [6,7,26], while perioperative chemotherapy, particularly FLOT, achieved consistent systemic disease control in adenocarcinoma (AC) [10,12,39].

The integration of immunotherapy into neoadjuvant frameworks has reshaped the therapeutic landscape. Trials such as PALACE-1 and PERFECT reported pCR rates approaching 50%, significantly higher than conventional CRT [18,21]. These outcomes align with immunotherapy successes in advanced settings (e.g., KEYNOTE-590 and CheckMate 648) [17,25,27,40], supporting the notion that checkpoint inhibition enhances tumour regression through immune-mediated cytotoxicity. Additionally, studies have shown that biomarkers such as PD-L1, HER2, and MSI status may influence treatment response and survival [13,41,42,43,44,45], reinforcing the need for biomarker-guided patient selection in future neoadjuvant trials.

The findings underline the importance of histological differentiation in treatment strategy. SCC, characterised by higher radiosensitivity, benefits most from CRT, achieving mean pCR rates around 35–45% and superior 5-year OS [15,16,46]. On the other hand, AC, more chemo-sensitive, responds better to perioperative CT, particularly FLOT, which offers durable systemic control despite lower pCR rates (20–30%) [10,47,48,49,50]. Such histology-driven divergence underscores the necessity for personalised treatment approaches, as emphasised by contemporary clinical guidelines [32,35,51].

Despite consistent trends, notable heterogeneity persists among studies. Clinically, differences in tumour location (oesophageal vs. GEJ), radiotherapy dose, chemotherapy regimen, and timing of surgery contribute to variable outcomes [7,8,52,53,54,55,56]. Methodologically, inconsistency in TRG systems (Mandard, Becker, CAP), follow-up duration, and study design affects reproducibility [33,34,36,57]. The lack of global standardisation in pathological reporting remains a major limitation in cross-study comparisons, a gap also noted by Kitagawa et al. (2024) and Luijten et al. (2023) [32,36].

Beyond classical pathology, molecular profiling offers new avenues for interpreting regression and survival. Emerging evidence suggests that genomic alterations such as CCNE1 amplification or MMR deficiency correlate with distinct regression patterns and prognosis [12,30,58]. Similarly, functional optimisation through prehabilitation or nutritional interventions may indirectly enhance response and recovery [28,59,60,61,62]. These insights highlight that both tumour biology and host physiology shape therapeutic success.

The principal limitation of this review is the methodological heterogeneity of the included studies, encompassing retrospective designs, variable TRG criteria, and incomplete biomarker reporting. Furthermore, the exclusion of non-English studies and grey literature may introduce selection bias. Although formal meta-analytic synthesis was not performed due to heterogeneity, the qualitative evidence strongly supports the prognostic relevance of pathological response.

Moving forward, the field must pursue standardised assessment of TRG and pCR, integrated with molecular and immunological profiling. The development of multicentre, biomarker-driven trials will be crucial to validate prognostic endpoints and refine risk stratification. As immunotherapy and targeted strategies gain momentum, the future of EGC management lies in personalised, multimodal, and data-driven therapeutic algorithms that align pathological regression with durable survival benefit.

## 5. Conclusions

This systematic review demonstrates that pathological response after neoadjuvant treatment is a strong and consistent predictor of survival in patients with oesophagogastric cancer (EGC). Across sixty contemporary studies published between 2020 and 2025, higher rates of pathological complete response (pCR) and major tumour regression (TRG 1–2) were consistently associated with improved overall and disease-free survival, regardless of study design or region.

Chemoradiotherapy (CRT) achieved superior local tumour control and higher pCR rates, particularly in squamous cell carcinoma (SCC), while perioperative chemotherapy (CT) provided greater systemic disease control in adenocarcinoma (AC) of the gastroesophageal junction (GEJ).

Emerging immunotherapy-based combinations (IO-CRT and IO-CT) showed the most encouraging early results, achieving pCR rates approaching 50% and suggesting potential paradigm shifts toward biologically driven multimodal strategies.

In conclusion, a deeper understanding of pathological response as both a prognostic and predictive marker can refine postoperative risk stratification, guide adjuvant therapy decisions, and inform the design of future precision-based treatment paradigms in oesophagogastric oncology.

## Figures and Tables

**Table 1:** Distribution of neoadjuvant treatment modalities represented in the included literature (2020–2025).

Study/First Author (Year)	Country	Study Design	Neoadjuvant Treatment Type	Specific Regimen(s)	Main Study Focus	Key Observations on Treatment Modality
van Laarhoven et al. (2025) [6]	Netherlands	Nationwide cohort	CRT (standard CROSS)	Carboplatin–paclitaxel + 41.4 Gy RT	Real-world validation of CROSS	Confirmed reproducible pCR rates (23%) and survival advantage in pCR group.
Sihag et al. (2022) [11]	USA	Retrospective cohort	CRT	CROSS	Trimodality therapy outcomes	CRT produced pCR 22%; pCR strongly associated with improved 5-year OS.
Heran et al. (2023) [12]	France	Retrospective cohort	CT (perioperative)	FLOT (fluorouracil, leucovorin, oxaliplatin, docetaxel)	Impact of MMR deficiency	CT regimen produced TRG-based regression; MMR-deficient tumours showed improved regression and survival.
Sun et al. (2021) [17]	China	Retrospective clinical study	CRT (RT-based)	3D-CRT/IMRT	Local control & survival	CRT improved 5-year local control rates; higher pCR in IMRT arm.
Li et al. (2021) [18]	China	Phase II clinical trial (PALACE-1)	CRT + Immunotherapy	Pembrolizumab + CRT (carboplatin–paclitaxel + 41.4 Gy)	Feasibility and efficacy	Combined approach yielded pCR 55%; 1-year OS 89%.
van den Ende et al. (2021) [21]	Netherlands	Phase II feasibility (PERFECT)	CRT + Immunotherapy	Atezolizumab + CROSS-based CRT	Resectable adenocarcinoma	Safe and feasible; pCR ≈ 30%, 1-year DFS 78%.

CT = chemotherapy; CRT = chemoradiotherapy; IO = immunotherapy; SCC = squamous cell carcinoma; GEJ = gastro-oesophageal junction; OS = overall survival; DFS = disease-free survival; pCR = pathological complete response; TRG = tumour regression grade. Note: Doki et al. (2022) [27] [CheckMate 648] is cited in Table 1 as contextual background only. This trial investigated nivolumab-based combination immunotherapy in advanced/metastatic oesophageal squamous cell carcinoma and did not involve neoadjuvant treatment; it therefore does not meet the eligibility criteria of this systematic review.

**Table 2:** Assessment systems.

Study/First Author (Year)	Regimen Type (CT/CRT/Other)	Response Assessment (pCR/TRG System)	pCR or Major Response (%)	Survival Outcomes (OS/DFS)	Key Findings/Observations
Allen et al. (2022) [28]	Prehabilitation during neoadjuvant	N/A	N/A	Post-op/fitness outcomes (NR)	Pilot RCT: better physiological reserve during neoadjuvant may enable treatment completion, indirectly favouring higher regression and survival potential.
Wu et al. (2025) [29]	Multimodal (CT ± IO; molecular profiling)	TRG (reported within molecular strata)	Reported in study (NR here)	OS stratified by molecular features (NR)	Multi-omic correlates of early-onset EGC: molecular subtypes associate with depth of regression and survival supports biomarker-driven selection.
Rustgi et al. (2024) [30]	Systemic therapy context (CCNE1 amplified)	N/A	N/A	OS signals by genotype (NR)	CCNE1 amplification linked to poorer outcomes; may predict reduced regression to standard perioperative CT, justifying alternative intensification.
Pucher et al. (2025) [31]	Early EGA (surgical pathology focus)	Nodal involvement (not TRG)	N/A	Prognosis via nodal status	Demonstrates prognostic weight of nodal disease; complements evidence that pathological downstaging (incl. nodes) underpins survival gains post-neoadjuvant.

AC = adenocarcinoma; CCNE1 = Cyclin E1 gene amplification; CRT = chemoradiotherapy; CT = chemotherapy; DFS = disease-free survival; EGC = esophagogastric cancer; FLOT = 5-Fluorouracil, Leucovorin, Oxaliplatin, and Docetaxel regimen; GEJ = gastroesophageal junction; IO = immunotherapy; N/A = not applicable; NR = not reported; OS = overall survival; pCR = pathological complete response; RCT = randomised controlled trial; TRG = tumour regression grade.

**Table 3:** Assessment systems used for Tumour Regression Grade (TRG) or Pathological Complete Response (pCR).

Study/First Author (Year)	Country	Cancer Type/Histology	Assessment System Used	Definition/Key Criteria	Application/Comments
Patruni et al. (2023) [33]	USA	Oesophagogastric (clinical practice)	CAP or Becker referenced	CAP 0 = pCR; 1–3 = increasing residual tumour	Advocates harmonised TRG selection by histology (Becker for AC; Mandard/CAP for CRT studies).
Kamarajah et al. (2022) [34]	UK/Netherlands/USA	Oesophagogastric (surgical techniques)	CAP referenced	ypT0N0 = pCR; 0–3 response scale	Methods paper on robotic surgery cites CAP for consistent pathology endpoints across centres.
Mine et al. (2022) [35]	Japan (multicentre)	GEJ cancers (postoperative outcomes)	pCR (ypT0N0) referenced	pCR = absence of viable tumour in resection specimen and nodes	Prospective registry notes pCR definition; TRG system variably used across centres.
Luijten et al. (2023) [36]	Netherlands	Oesophagogastric (MDT practice)	CAP recommended in MDT	Standardised 0–3 response for reporting	Study of MDT variation recommends declaring a single pathology framework (CAP) for comparability.
Nakamura et al. (2024) [37]	Brazil/Japan	Oesophageal & EGJ (biomarkers)	TRG referenced as outcome	Major vs. minor regression per study TRG	Meta-analysis on HSP prognostics stratifies outcomes by reported TRG categories.
Hiki et al. (2025) [38]	Japan	GEJ cancer (APG surgery)	CAP (surgical pathology)	pCR and margin status per CAP	Technique paper uses CAP conventions for pathological reporting in GEJ resections.

AC = adenocarcinoma; AJCC = American Joint Committee on Cancer; APG = appetite-preserving gastrectomy; CAP = College of American Pathologists; CRT = chemoradiotherapy; CT = chemotherapy; EGJ/GEJ = (gastro-)oesophageal junction; HSP = heat shock protein; MDT = multidisciplinary team; NCCN = National Comprehensive Cancer Network; OS = overall survival; pCR = pathological complete response; SCC = squamous cell carcinoma; TRG = tumour regression grade.

## Data Availability

The original contributions presented in this study are included in the article/Supplementary Materials. Further inquiries can be directed to the corresponding authors.

## Supplementary Materials

The following supporting information can be downloaded at: https://www.mdpi.com/article/10.3390/diagnostics16162524/s1, File S1: PRISMA 2020 Checklist.

## Abbreviations

The following abbreviations are used in this manuscript:

AC	Adenocarcinoma
AJCC	American Joint Committee on Cancer
CAP	College of American Pathologists
CCNE1	Cyclin E1 gene
CRT	Chemoradiotherapy
CT	Chemotherapy
DFS	Disease-Free Survival
EGC	Esophagogastric Cancer
FLOT	5-Fluorouracil, Leucovorin, Oxaliplatin, and Docetaxel
GEJ	Gastroesophageal Junction
HER2	Human Epidermal Growth Factor Receptor 2
HR	Hazard Ratio
IMRT	Intensity-Modulated Radiotherapy
IO	Immunotherapy
IO-CRT	Immunotherapy combined with Chemoradiotherapy
IO-CT	Immunotherapy combined with Chemotherapy
MMR	Mismatch Repair
MSI	Microsatellite Instability
NOS	Newcastle–Ottawa Scale
NR	Not Reported
OS	Overall Survival
pCR	pathological complete response
PD-1/PD-L1	Programmed Cell Death Protein 1/Ligand 1
PRISMA	Preferred Reporting Items for Systematic Reviews and Meta-Analyses
R0 Resection	Microscopically Margin-Negative Resection
RCT	Randomised Controlled Trial
RT	Radiotherapy
SCC	Squamous Cell Carcinoma
TRG	Tumour Regression Grade

## Appendix A

**Table A1:** Search strategies and applied filters (2020–2025).

Database	Search Strategy/Query Syntax	Filters and Limits Applied
**PubMed (MEDLINE)**	(“esophageal neoplasms”[MeSH] OR “esophageal cancer” OR “oesophageal cancer” OR “gastroesophageal junction cancer” OR “gastric cancer”) AND (“neoadjuvant therapy”[MeSH] OR “preoperative chemotherapy” OR “chemoradiotherapy” OR “neoadjuvant chemoradiotherapy” OR “perioperative chemotherapy”) AND (“pathologic response” OR “tumor regression grade” OR “TRG” OR “pathological complete response” OR “pCR” OR “survival” OR “outcome”)	English language; Humans; Publication date from 1 January 2020 to 20 September 2025
**Scopus**	TITLE-ABS-KEY (“esophageal cancer” OR “oesophageal cancer” OR “gastroesophageal junction cancer” OR “gastric cancer”) AND TITLE-ABS-KEY (“neoadjuvant chemotherapy” OR “chemoradiotherapy” OR “preoperative therapy” OR “perioperative chemotherapy”) AND TITLE-ABS-KEY (“pathologic response” OR “tumor regression grade” OR “pCR” OR “survival”)	English; Humans; 2020–2025; Article or Review
**Web of Science (Core Collection)**	TS=(“esophageal cancer” OR “oesophageal cancer” OR “gastroesophageal junction cancer” OR “gastric cancer”) AND TS=(“neoadjuvant therapy” OR “chemoradiotherapy” OR “preoperative chemotherapy”) AND TS=(“pathologic response” OR “tumor regression grade” OR “pCR” OR “survival”)	Document type: Article or Review; Language: English; Timespan: 2020–2025
